# Remarks on Medical Reform

**Published:** 1813-03

**Authors:** 


*To the Editors of the Medical and Physical Journal.*

GENTLEMEN,

SITUATED beyond the "Welch mountain," your Number for January has but just reached me. The strictures contained in your Half-yearly Report of the Progress of Medicine, upon the Committee of Apothecaries and the business upon which they are assembled, I hope will call forth some abler pen. In the mean time, as you have stated in the latter part of your Number that your pages are open "to liberal and candid animadversions," I trust they will not be closed against mine, although I do not occupy a very high station in the temple of Æsculapius. My observations will be drawn from simple and notorious facts. Being hastily put together, they may contain some inaccuracies and boldness of expression, but which, I hope, may be excused, if not justified, by your own comments.

I must pass over some pages to arrive at that part where you assert that "already it has gone abroad that trade rather than science is the object of the committee." Now, gentlemen, if such a report has gone abroad, it is more than we were aware of in this part of the United Kingdom. We never did conceive that the committee, which we know is made up of "mixed practitioners," was acting for the benefit of the

---

Apothecaries' Company, or that it had any connection with it. I am certain the report mentions nothing of the kind, and from the report only, which professes to be a statement of the proceedings of the committee, and the views of the apothecaries, can we judge. I much fear, if "the Demon of Discord" has gone forth, the author of the "Half-yearly Report" has been the first to sound the horn from "the Welsh mountain."

I cannot but agree, though I lament, that the profession labors under much ridicule and contempt, probably more in this country than in any other. It may arise, in some measure, no doubt, from the reception of improper members; but I am inclined to believe that it is much more owing to jealousy and dissention. Every profession is more or less intruded upon by improper persons. It is a universal complaint here and in every country. As to charlatans, or open quacks, they do some injury; but they enjoy their day, and soon sink to their proper level. It is only by noticing them that they are elevated into view: let the faculty treat them with the silent contempt they deserve, and they soon fall into their original obscurity; or, if their arts are really pernicious to society at large, let the legislature notice it. Indeed I am not hazarding too much when I state that this has been the policy of the Royal College of London. I believe one of the duties originally imposed upon that learned body was to prevent or check the growth of quackery. This we know they have long since ceased to do, and, if such an ordinance is contained in their charter, it has become a dead letter.

The author of the Half-yearly Report asks, "who are the apothecaries of Great Britain?" and, after much disquisition, informs us that "they neither range under the banners of Pharmacopola Verus, the true Surgeon, nor the Physician."* There need no ghost from the dead to tell us this: the whole community know it; every shopkeeper in England and Wales are aware of it; and to confound, for an instant, the interests of the "mixed practitioner" with the Apothecaries' Company, is a waste of time. Granted, the simple term Apothecary is a misnomer. But to what does all this tend? Is it to soften down the threat held out in different parts of this

* Before it can be believed that the *apothecary* does *not* range under the banner of *Pharmacopola Verus,* some evidence more convincing that even that of the ghost must be produced. By confounding one part of the Report with another at a distance, W. A. D. has applied a remark to the *apothecary,* which the Report applies to the *mixed practitioner.*—Editors.

this paper, that the apothecaries will be opposed in their proposed application to parliament by the united strength of the London Colleges of Physicians and Surgeons? Unless the College of Surgeons are governed by an aristocracy, under the control of the College of Physicians, and which I am not disposed to believe, much opposition need not be apprehended from that quarter. We know that the privileges of the College of Surgeons are open to every man *regularly educated* in the profession, and competent to pass an examination in anatomy and operative surgery; furthermore, it is well known, that, with very few exceptions, every "mixed practitioner" is a member of the College of Surgeons. The privileges of the Royal College of Physicians are more exclusive. The London temple of medicine is more strongly guarded, and fenced with every species of learned and chartered fortification. We are told, in a tone of defiance, by the reporter, that "the College of Physicians of London has a charter, considered to be fully equal to all its own particular and individual purposes;" and, lest the "mixed practitioners" should doubt of its adequacy to its own defence, they are farther assured that "every measure that touches, however lightly, on their chartered rights, will be resisted; and *effectually resisted*." But, not content even with this defiance and assertion, the author goes on to tell the poor forlorn apothecaries, in a subsequent paragraph, "that the two colleges will feel every attack upon their privileges like a wound;" and "they will resist the efforts of the Committee with all the power that *influence*, *opinion*, and *talent*, confers." Then, seemingly enjoying the apparent discord which is said to have taken place, the little phalanx of apothecaries is overwhelmed, literally, in the idea of the writer, and sent back to their pestles and mortars. But no, "the strong arm of the country practitioners" will march to the support of their brethren, and then let those who have attempted "to spread the leven of apprehension to the remotest nooks of the island, look to their own particular condition."

I know that I am treading upon tender ground, but far be it from me to throw out one assertion, or even one word, with intentional disrespect against that learned and venerable body, the Royal College of Physicians of London. I respect and reverence every institution of our forefathers, and none more than the College, which I know was instituted for the wisest and best of purposes; but I am here answering an individual, whether of the Royal College, or not, I am ignorant; and, without disrespect to any individual belonging to it, I think it may be shewn that the fellows of the London college are not the only persons in England and Wales who possess a competent knowledge of physic. Indeed the author of the Half-yearly Report has conceded more than he seems to be perfectly aware of or intended, for, after driving the surgeon-apothecary "from the banners of Pharmacopola Verus, the true Surgeon, or the Physician," he states that they, "the mixed practitioners," are a body distinct from either, *but combining the energies, and exercising the functions, of the three.* The mass of the population is submitted to their care, with few exceptions indeed. This circumstance alone, were it not a fact that *they have generally a most extensive view of the whole system of medical practice*, will mark them as of great consideration to a wise government. Now I say this is placing the "mixed practitioner" in a more elevated situation than he could have hoped for, or his utmost ambition would have led him to assume. It is a formal acknowledgment of his public utility; then why not, in justice, go a step further, and acknowledge also that the mixed practitioner likewise possesses the confidence of the mass of the population? Why, in the following paragraph, degrade this useful member of society by saying "that a law to protect the rights, and define the privileges, of persons incapable of performing the functions of the station into which they have obtruded, is an absurdity." To pass a law with such a preamble would certainly be absurd; but, having asserted this calumny, it behoved the reporter to bring forward some proofs that the persons he had previously passed such an eulogium upon, are incapable. I assert that the "mixed practitioners" draw from the same springs, that they have their medical and chirurgical knowledge from the same fountains, the same schools, and the same sources, as the physicians and surgeons of the London colleges. The "mixed practitioner" who has received his medical education in London or Edinburgh, has received it from the same professors as the physician of the Royal College: he has studied the same authors, and the volumes of Hippocrates and Celsus, down to Cullen and Brown, are equally open to the perusal of the surgeon-apothecary as to the possessor of a diploma. Let me refer to the volumes of this useful work from its commencement to the present day, and I venture to state, without danger of contradiction, that many of the best papers in medicine, surgery, and pharmacy, have been received from the "mixed practitioners."

I will now ask the reporter to whose medical charge have the government thought fit to confide the health of the mass of the sailors and soldiers of the nation? The army and navy lists will shortly inform us to "mixed practitioners," persons persons combining the energies, and exercising the functions, of the three divisions. The few among those respectable gentlemen who add M. D. to their names, have mostly received their degrees from the Scotch universities. It is true the rank of Physician to the Forces still continues, and the majority of that particular rank, from courtesy and former custom, are still selected from the London College. I cannot, however, enter more particularly into this part of the subject without bringing an old controversy upon the *tapis*, which is far from my intention. It is sufficient for my purpose to shew that the able and confidential medical officers in the army and navy are from the "mixed practitioners." It would be easy to select numerous instances of splendid talent from amongst those gentlemen; but, where merit is so generally diffused, it might be an invidious task for an obscure individual. The national records and lists are sufficient and glorious evidence. I have myself had the honor of serving in India many years. The Indian army, including a body, I presume, of upwards of 100,000 men, are solely confided to "mixed practitioners." There are many physicians in the medical department of the Company's army, but physician forms no particular rank, and the right of adding M.D. to the surname neither adds to the respectability of the persons, or increases the confidence of the respectable society of which they form a part. There is no place in the British empire where the faculty enjoy more confidence or esteem than they do in India, London not excepted, and I will add there is no place where they have shewed themselves more worthy.

With regard to the medical practice in England and Wales, it is sufficiently shewn and conceded by the reporter, that the mass of the population are under the care of the mixed practitioner. Physicians of the college are to be found in plenty in the metropolis, and wherever wealth abounds; but the inferior towns, villages, and hamlets, of the kingdom, as well as the middling and lower ranks in London, are left to the surgeon-apothecary. The situation of the surgeons of the army and navy has been within a few years past greatly improved, both in emolument and respectability, by the Government; and why is not the same deserving person in civil life, the active drudge of the profession, who enjoys the confidence of the majority of the nation, to endeavor to improve his station in progress with the society to whom he is so useful? Is fear of impinging the chartered privileges of any particular body to interfere with the rights of a whole and numerous profession, and, what is more, for the public good? Let those who are so

 tenacious tenacious of their own privileges, be extremely careful how they censure another body for want of integrity. Will it not be inquired, for whom they are defending those vaunted privileges? Whether for the public good or individual benefit? If it can be shewn that the *medical education* of the mixed practitioner is identically the same as that of the physician, with the exception of passing a certain period at Oxford or Cambridge, where every knowledge but medical knowledge is to be acquired; I say, if this can be proved, it is not their charter, their influence, opinion, or talent, that will prevent a useful member from assuming his proper station in society, or from receiving a just remuneration for his labor.

I have stated that, among the medical officers of the army and navy, and the East-India Company's service, there are many who have the degree of doctor of physic. These degrees are chiefly from the northern universities. Why is this so? Surely there must be something wrong here, or why is a man, after having passed various examinations, and who is judged fit and proper to be entrusted with the lives of the defenders of his country, when he wishes to gain an honorary distinction in his profession; I say, why is he obliged to have recourse to universities at the very extremity of the island, and upon whom so much reproach has so frequently, and too often undeservedly, been thrown? But is it impossible to remedy this evil? Are the numerous gentlemen above-mentioned incapable of passing a *medical examination* before the college of London? It cannot be. Does their incapacity originate in having served an apprenticeship to a surgeon or an apothecary? Or is it, that they have not idled away a portion of their time in either of the English universities, where, as I before observed, every knowledge almost is to be obtained in perfection but medical? I submit, if there is any truth in the above statement, whether it is not almost time for the *public good* that these things should be inquired into and remedied.

I am, Gentlemen,

*South Wales,* Your obedient Servant,

*Jan.* 14, 1813. W. A. D.*

*For*

* We shall, probably, gratify the writer of the preceding paper by permitting him the contemplation of his lucubrations in print. It was not, however, for the gratification of W. A. D. in this point, that we allowed his paper to occupy a portion of our Journal: we have more cogent reasons. These are, the importance of the subject on which he writes—the opportunity it gives us to shew to the public the readiness with which we admit animadversions which bear with harshness on ourselves—and the means it affords of again stating to the medical faculty, particularly the country practitioners, under the denomination of surgeon-apothecary, how fully we are satisfied of the honorable intentions, and of the now able proceedings, of the London committee.

W. A. D. seems to have been altogether mis-informed of the state of public opinion on the primary proceedings of the London Committee of Apothecaries; nor does he appear to have apprehended the mischievous effects that would have operated on all its acts, if such opinions had been allowed to exist, without calling on the committee to exonerate itself of all suspicion. How could the enemies of the cause of the surgeon-apothecary have injured that cause more effectually than by bringing into discredit the conduct of the committee? If it had been generally understood that the object of the committee was the aggrandisement of the Apothecaries' Company, as had been often insinuated, the main force and power of the cause had been withheld. The country practitioners, under this circumstance, would have been little solicitous to assist their brethren in London; and the great object would have been lost by a disjunction of the powers that were to carry it into effect. In this state of public opinion the Report took up the subject, and shewed to the committee, in a way not to be disregarded, the necessity of a clear explication of the principles on which it acted. This the committee has done in a manner equally creditable to itself, and useful to the cause in which it is engaged. The consequence has been an entire removal of suspicion, and an ardent co-operation of the country practitioners. The effort now making will, we sincerely hope, be crowned with success; but we do not yet apprehend how far it will be supported by any colleges, or corporate bodies.

So far as W. A. D.'s observations apply to ourselves, we have but the following remark to make. He says, "why in the following paragraph degrade this useful member (the mixed practitioner) of society, by saying that a law to protect the rights, and define the privileges, of of persons incapable of performing the functions of a station into which they have obtruded, is an absurdity." It is evident that to protect by law persons in a station, the functions of which they are incompetent to perform, is an absurdity; but it is, perhaps, an absurdity to which the imperfections of all human institutions will compel us to submit. It is equally evident that, by " persons incompetent," the Reporter did not mean the aggregate mass of surgeon-apothecaries, but certain individuals, who, from the want of a proper barrier, have suddenly merged into surgeon-apothecaries, accoucheurs, &c. from being livery-servants, grocers, tinkers, and coblers. From the context it will appear that the Reporter always thinks highly of the OFFICE of the surgeon-apothecary, and of the individuals composing their body, when properly educated; but the surreptitious intrusion of the vagabonds just named, will always be reprobated. It is to be lamented that the new code of law must leave the cobler and the tinker recognised and protected in their assumed position, to mangle and to carve, until time shall call them to their account. Without being fully aware of the difficulty or inequity of *ex-post-facto* laws, and seeing only the crying evil, the Reporter may have put the case too strongly, but never to have the meaning the garbled statement of W. A. D. has given to it.—EDITORS.

